# Self-filling enclosures to experimentally assess plankton response to pulse nutrient enrichments

**DOI:** 10.1093/plankt/fbac074

**Published:** 2023-01-13

**Authors:** Pau Giménez-Grau, Lluís Camarero, Carlos Palacín-Lizarbe, Marc Sala-Faig, Aitziber Zufiaurre, Sergi Pla-Rabés, Marisol Felip, Jordi Catalan

**Affiliations:** CREAF, Cerdanyola del Vallès, Catalonia E-08193, Spain; Department of Bioscience, Aarhus University, Aarhus C DK-8000, Denmark; CEAB, CSIC, Blanes, Catalonia E-17300, Spain; CREAF, Cerdanyola del Vallès, Catalonia E-08193, Spain; Department of Environmental and Biological Sciences, University of Eastern Finland, Kuopio FI-70211, Finland; CREAF, Cerdanyola del Vallès, Catalonia E-08193, Spain; CEAB, CSIC, Blanes, Catalonia E-17300, Spain; CREAF, Cerdanyola del Vallès, Catalonia E-08193, Spain; área de Biodiversidad, Gestión ambiental de Navarra-Nafarroako Ingurumenkudeaketa (GAN-NIK), Pamplona-Iruñea, Navarra E-31001, Spain; CREAF, Cerdanyola del Vallès, Catalonia E-08193, Spain; CREAF, Cerdanyola del Vallès, Catalonia E-08193, Spain; Departament de Biologia Evolutiva, Ecologia i Ciències Ambientals, Universitat de Barcelona, Barcelona, Catalonia E-08028, Spain; CREAF, Cerdanyola del Vallès, Catalonia E-08193, Spain; CSIC, Bellaterra E-08193, Spain

**Keywords:** microcosms, mesocosms, nutrient additions, experimental plankton perturbations, phytoplankton, protists, lake field experiments

## Abstract

Experimental nutrient additions are a fundamental approach to investigating plankton ecology. Possibilities range from whole-lake fertilization to flask assays encompassing a trade-off between closeness to the “real world” and feasibility and replication. Here we describe an enclosure type that minimizes the manipulation of planktonic communities during the enclosure filling. The enclosure (typically ~100 L volume) consists of a narrow translucent cylinder that can comprise the entire photic zone (or a large part of it in clear deep lakes, e.g. 20-m long) and holds a sediment trap at the bottom for recovering the sinking material. The enclosures are inexpensive and straightforward to build. Thus, many can be used in an experiment, favoring the diversity of treatments and the number of replicates. They also are lightweight with easy transport and use in lakes that cannot be reached by road. The enclosures are fundamentally aimed at investigating the short-term response of the planktonic community, integrated across the photic zone, to pulse perturbations using before and after comparisons and multiple replication and treatments. The pros and cons of the enclosure design are evaluated based on experience gained in Lake Redon, a high mountain ultraoligotrophic deep lake in the Pyrenees.

## INTRODUCTION

Planktonic ecosystems commonly show episodes of higher productivity embedded within prolonged periods of reduced activity. This pattern is particularly discernible in oligotrophic ecosystems where phytoplankton growth increases during periods of water column mixing when nutrients are loaded into the photic zone from the deep layers (Sommer, 1986; Goldman, 1988; Domis *et al*., 2013). The study of these episodic enrichments is critical for the assessment of the long-term biogeochemical dynamics and the understanding of the ecology and evolution of planktonic organisms and their assemblage into communities (Harris, 1980; Falkowski *et al*., 1998; Mcandrew *et al.*, 2007; Spivak *et al*., 2011). One of the challenges of these studies is untangling the role of co-occurring driving factors (Richardson *et al*., 2017; Morabito *et al*., 2018). The nutrient enrichment coincides with a shift in various physical conditions, e.g. mixing depth, mixing intensity, light regime and temperature (Reynolds, 2006). Experimental additions are a way to disentangle the effects of targeted nutrients and other substances of biological influence (e.g. dissolved organic matter, vitamins, toxicants) from other environmental factors (Downing *et al*., 1999; Benton *et al*., 2007; Faithfull *et al*., 2011; Spivak *et al*., 2011). The available methods for nutrient enrichments cover a wide range of laboratory and field settings, from flask assays to whole-lake fertilization, each with its pros and cons, encompassing a trade-off between closeness to the “real world” and feasibility and replication (Riebesell *et al*., 2010). The common feature of the different methods is that they aim to manipulate an environmental gradient and explore its role in structuring communities and functional traits (Fraser and Keddy, 1997).

Designs of enclosures used in aquatic ecological research have been repeatedly reviewed (Parsons, 1982; Sanders, 1985; Scor-Working-Group-85, 1991; Riebesell *et al*., 2010; Stewart *et al*., 2013; Legendre *et al*., 2018). Enclosures of less than 1 L are limited to prokaryote and protist experiments, whereas 10–100 L are required to consider zooplankton dynamics and larger enclosures are necessary for higher trophic levels (Parsons, 1982). The Scor-Working-Group-85 (Scor-Working-Group-85, 1991) divided pelagic experimental enclosures into three size classes: microcosms (<1 m^3^), mesocosms (1–1000 m^3^) and macrocosms (>1000 m^3^). Microcosms provide more experimental control (i.e. replication, controllability and homogeneity) and lower cost, and macrocosms are closer to reality, capture better some biological complexity (e.g. trophic interaction, dispersal), and allow recurrent sampling (i.e. time series). In any case, the ability to deal with high trophic levels and approximate hydrodynamics found in nature is always limited (Riebesell *et al*., 2010). Trophic interactions are affected by the size of the experimental units (Marrase *et al*., 1992), and phytoplankton development appears more consistent across enclosures of different sizes than bacterioplankton and zooplankton (Kuiper *et al*., 1983).

Episodic nutrient enrichments in lakes occur at temporal scales of days to weeks. Therefore, hypothesis testing concerning the response of primary production, physiological traits, and community processes, up to protist level, to nutrient additions can be adequately performed using multiple enclosures (>10) of relatively small size (50–1000 L) during days to weeks (Riebesell *et al*., 2010). Here, we describe an enclosure system for *in situ* lake experiments of a relatively short duration that minimizes the manipulation of the planktonic community during the enclosure filling and allows for many replicates at a low cost. The enclosures are lightweight and folded for transportation, so they can be used in remote locations without road access and deployed using small boats. Although the method to enclose a water volume by lifting and unfolding a bag has been previously used—e.g. with the aid of divers (Menzel and Case, 1977)—the system we describe is straightforward and valuable for perturbation experiments of a few days to weeks.

The experience gained during the “Episodic Nutrient Enrichment Experiment (ENEX)” in Lake Redon, an ultraoligotrophic deep high-mountain lake in the Pyrenees (Ventura *et al*., 2000), is used to illustrate the suitability and limitations of the enclosure system described, and discuss the possibilities of alternative designs that could be applied in other systems following the same general design concept. The planktonic community in Lake Redon is P-limited due to the long-range atmospheric transport of reactive nitrogen (Camarero and Catalan, 2012). The primary objective of the ENEX experiment was to evaluate the effects of the fluctuations in P availability and N:P imbalance on the structure of the planktonic protist community (Zufiaurre *et al*., 2021) and its stoichiometry (Giménez-Grau *et al*., 2020). We first describe the enclosure design and use as performed in the ENEX experiment, and, in the discussion, we comment on alternative options and designs according to other objectives and lake characteristics.

Ecological field experiments are inevitably subject to trade-offs between what is desirable and what can be afforded. Whatever the size of the experiment, replication is a critical issue. The lower the cost, the higher the potential number of replicates and treatments and, thus, more robust results (Riebesell *et al*., 2010). The enclosure described here is low cost (in our case, ~60€ per enclosure), simple in its components and deployment, and highly reliable in results.

## METHOD

### Enclosure design

**Fig. 1:**
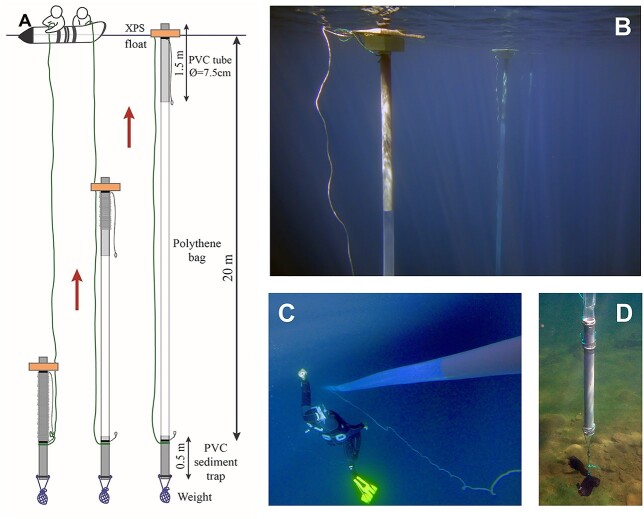
(**A**) Enclosure sketch, deployment and filling method. Underwater view of the upper (**B**), middle (**C**) and bottom (**D**) parts of the enclosures.

The enclosures are constructed using tubular-shaped clear polythene bags (diameter: 8.5 cm; length: 20 m) and two polyvinyl chloride (PVC) tubes, attached one at each extreme of the bag (Fig. 1). The PVC tube at the lower end (length: 0.5 m) is closed with a sealed stopper and serves as a sediment trap, while the upper tube (length: 1.5 m) enables the gaseous exchange with the atmosphere, prevents the bag from collapsing, and facilitates the attachments. An extruded polystyrene float is attached to this upper tube to hold the enclosure at the water surface. A weight is tied to the sediment trap to stretch the bag and help the enclosure sink during deployment. Each enclosure is self-filled (see below) with ~100 L of water from 0 to 20-m lake depths. The complete list of items and some extra details necessary for the enclosure assembly are provided in Table I. Enclosures cannot be reused without changing the polythene bags and cleaning the PVC tubes.

**Table I:** Items, materials and guiding comments to build, deploy and use the enclosure system described

Item	Materials	Function	Comments
Upper holding tube	PVC, 7.5 cm Ø, 1.5 m long	Tube for attachment of the flexible bag, floats, rope and mooring connection. It acts as the mast for the bag during deployment.	The tube length depends on the bag’s length since it acts as a pole to fold the bag around during deployment. The diameter must be adapted to the bag diameter. Transparent materials can be considered (for example, methacrylate)
Tube bag roll	Polythene, 8.5 cm Ø, 20 m long	Defines the enclosure growth environment	The polythene bags are sold in rolls of many meters (e.g. 200 m) and are available in many diameters, commonly from 4 to 30 cm, allowing for alternative designs without specific customer manufacturing demands.
Lower sediment trap	PVC, 7.5 cm Ø, 1.5 m long. Sealed tap bottom	Collect the sinking material. Attachment for the guiding rope and weight net bag.	A funnel connecting the bag and tube should be introduced if the bag diameter is enlarged.
Floats	Extruded polystyrene. Size tailored to the weight of the enclosure and lake wind conditions.	Maintain upraising buoyancy	They have to be adapted to the upper tube diameter. Two sets are used. One is located below the rope attachment, used for uprising during deployment. Another is above the clamp attachment, added after filling the enclosure to increase buoyancy.
Clamps	Stain steel. Adapted to PVC tube diameter	They hold together the bag and tubes and are the attachment points of the rope and weight net bag.	
Net bag	Any suitable material	Bag for extra weight.	Depending on the transport facilities, weight can be local material (e.g. littoral rocks) or specific items of known weight.
Rope	Non-elastic material	It keeps the system folded before deployment. Controls the deployment from the boat, and the upper part is released once the desired depth is reached	
Addition and sampling tubes	Plastic tubes. 20-m long. About 1 L volume. With taps at the extremes.	Initial nutrient addition (1 tube) and final sampling (5 tubes).	Taps are necessary to keep the nutrient solution within the tube during the introduction in the enclosure. No need for sampling.
Fishing sinkers	Lead and stainless steel.	Used for sinking the nutrient addition and sampling tubes.	
Vacuum flask	Polypropylene or glass.	For emptying the sampling tubes by siphoning vacuum.	
Vacuum pump	Hand- or battery-operated with gauge	Producing vacuum in the sampling flask.	Keep pressure as low as possible to maintain a sufficient flow, and only pump the tube volume for a balanced water column sampling.

ENEX enclosure dimensions and features are indicated with comments on some alternative options.

### Enclosure deployment

The deployment of the enclosure consists of the following steps. (i) The enclosure is assembled: the polythene bag is folded around the upper tube, and this tube is held together with the sediment trap by a rope lacing using an escape knot (Fig. 1A; Supplementary Information Video, 0:19 min). (ii) The folded enclosure is placed horizontally on the lake surface and filled with water (Supplementary Information Video, 1:05 min). (iii) The folded enclosure is allowed to sink until the depth equivalent to the maximum length of the enclosure (20 m in the case described here), where the rope stops it (Supplementary Information Video, 1:45 min). (iv) When the rope tensions, the lacing, which holds the tubes together, unties (Supplementary Information Video, 0:45 min). The upper tube starts to float toward the lake surface, thus gently filling the bag with water from the upper 20 m of the water column (Fig. 1A; Supplementary Information Video, 2:14 min). (v) Finally, extra floats are added to the upper part to increase the buoyancy (Supplementary Information Video, 2:38 min).

### Nutrient addition

Once the enclosure is filled with lake water and attached to the mooring, one may proceed to the specific addition of the planned treatment. A 20-m long thin plastic tube is filled with ~0.9 L of nutrient-enriched water. The tube is introduced inside the enclosure and is dropped to the bottom with the help of weight at the front end (Supplementary Information Video, 2:58 min). The tube is open in the front end and closed at the back end using a tap so that it does not leak its content during the deployment within the enclosure. Once the enclosure’s bottom is reached, the tube’s rear end at the surface is opened. The solution is released homogeneously along the enclosure’s water column as the tube is constantly withdrawn from the enclosure (Supplementary Information Video, 3:14 min). Finally, the enclosure is attached to a rope that holds it to an all-enclosure mooring (Supplementary Information Video, 3:37 m). The enclosure deployment and nutrient additions can be performed from a small boat without problems.

### Mooring

The enclosures are individually hung from a line of a mooring system, maintaining a distance between them sufficient to minimize direct shadowing (Fig. 1B). An east–west alignment of the mooring system guarantees equivalent exposure of each enclosure to solar irradiance. Placement needs to consider also potential surrounding relief shadows. Depending on the lake dimensions, the mooring system can consist of buoys and anchor weights or be attached to existing platforms or the shore. The weight at the base of each enclosure should be sufficient to avoid vertical deflection of the enclosure during windy situations. Using a net bag and on-site stones allow adjusting the necessary weight without transporting extra weights to the site (Fig. 1D). If strong currents are expected, more buoys and anchor weights interspersed between every few enclosures can be added. In Lake Redon, winds can be relatively strong, but the fetch is short, we were using only mooring at the extremes, and the line sustained 22 enclosures (Giménez-Grau *et al*., 2020).

### Enclosure final sampling

These enclosures should be considered microcosms (<1 m^3^) according to the Scor-Working-Group-85 (Scor-Working-Group-85, 1991) classification. Intermediate sampling during the experiment will cause too much disturbance in the relatively small volume (~100 L). At the end of the experiment, an integrated (0–20 m deep) water sample can be obtained from each enclosure by sinking 20-m long plastic tubes with weight. In the ENEX experiment, we used five clean tubes, each of 1-L capacity, deployed simultaneously. The integrated sample was recovered by pumping the exact volume retained in each deployed tube into a vacuum flask using a hand pump to reduce potential cell damage (Table I). Once the water column part is sampled, the enclosure is uplifted gently until reaching the sediment trap. The sediment trap is detached from the bag, and its water content is collected and kept for later filtration in the laboratory.

## DISCUSSION

### Experimentation in difficult-to-reach sites

Large experimental enclosures in lakes require transport and operational facilities that are usually only available in well-connected areas. Large mesocosm experiments have been typically carried out not far away from limnological laboratories [e.g. Belham Tarn, England (Lack and Lund, 1974); Lake Biwa, Japan (Nakano *et al*., 2001)]. When the experiments are performed across many sites (Scharfenberger *et al*., 2019) or in locations difficult to reach (Halac *et al*., 1997), the enclosure volume quickly declines to smaller volumes, and replication is limited. If field experimentation is too difficult, water samples are brought to labs, and flask assays are performed (Jacquemin *et al*., 2018). In the case of the ENEX experiment in Lake Redon, located in an alpine mountain landscape without access by road or easy tracks, all the material was transported in backpacks. Even with that restriction, 22 enclosures were deployed. Therefore, one of the strengths of the described system is the affordability of an elevated number of treatments and replicates even in remote locations.

### Experiment duration

The enclosure system described has been thought of as a way to perform short perturbation experiments on the planktonic community. Colonization is excluded, and the response depends on the existing species assemblage enclosed during deployment. Therefore, the duration of the experiment has to be tailored to the response capacity of the community. Ideally, the investigation will focus primarily on growth responses, thus avoiding decay phases and secondary successions within the enclosure. The amount of material collected in the sediment trap compared with the material remaining in the water column of the transparent bag will inform about the growth phase of the community. However, this will be a piece of information gathered at the end of the experiment. Alternatively, a fluorescence sensor can be installed in one enclosure where higher growth is expected. Another option is to dedicate an extra enclosure for determining the end of the experiment, in which fluorescence profiles are performed regularly to follow the temporal sequence of the response. According to the objectives, this enclosure should be treated with the expected more informative additions. However, it should not be used in the intercomparison of the treatments, as profiling will introduce a highly artificial mixing in the enclosure.

In extremely oligotrophic cold systems such as Lake Redon, the duration can last several weeks without significant decay of the responding populations. In the ENEX experiment, the enclosures were deployed on 5–6 August 2013 and recovered 25 days later. Given the starting low populations in the epilimnetic waters and the relatively low-temperature conditions (14–6°C), a few weeks were a reasonable approach (Parsons, 1982). Shorter experimental durations are expected in warmer and more productive locations. In highly productive systems, the experiment should last from a few hours to a few days. The most complicated systems could be those that experience large periodical fluctuations in productivity. They may show extremely clear waters but hold a community capable of significant and rapid blooms, like some marine systems (Estrada *et al*., 1988). Because of the low cost of the enclosures, a preliminary test for adjusting the time of the experiment is recommended.

### Sampling and measurements

The enclosure design is conceived to integrate changes across the epilimnetic or photic zone gradients. The tubes’ system described above delivers 5 L of an integrated sample, which allows for many measurements, but other volumes can be considered, including pumping the entire enclosure. For instance, in the ENEX experiment, we measured in each enclosure: total dissolved phosphorus (TDP), NH_4_^+^, NO_2_^−^, NO_3_^−^; dissolved organic carbon (DOC), particulate C (PC), particulate N (PN), particulate P (PP), chlorophyll a (Chla) and other pigments by liquid chromatography, protists abundance, and biovolume, and the prokaryote abundance estimated as 4′,6-diamidino-2-fenilindol stain (DAPI) counts. The same biogeochemical variables were measured in the sediment traps, and diatom valves and chrysophyte cysts were assessed. The critical point is to maintain top-to-bottom integrated water samples, as the heterogeneity within the enclosure may be vertically substantial. The method’s strength is comparing treatments rather than a time series of changes within the enclosure. The initial conditions must be assessed with a similar integrated sample of the lake water column at the beginning of the experiment. Comparing the initial and final conditions provides information about rates as the system is basically closed to dissolved and particulate matter exchanges (Zufiaurre *et al*., 2021).

### Coherence and replication

The enclosure’s filling method is quite friendly for the organisms as they are not submitted to any particular stress (e.g. light, temperature, pressure), and the vertical heterogeneity in the photic zone is fully captured during the regular and slow-pace filling. There is an initial community representative of the photic zone in each enclosure. Enclosure and incubation systems that require pumping or weighted integration of samples in containers may introduce initial biases (e.g. high mortality of abundant but sensitive organisms) compared with the self-filling system used here (Riebesell *et al*., 2010).

The main power of experimental approaches is reducing the causal effects to those manipulated by the experimenter. Unfortunately, field experiments can hardly follow the optimal experimental designs equivalent to those obtained in a lab. The ENEX experiment took about 12 h to deploy and perform the additions in the 22 enclosures. The relatively long-lasting and self-filling system involves some risk of increasing the variation in initial conditions. The enclosures are not filled from a homogenized common pool and could cause noisy responses to the treatments. It is assumed that the treatment effects would be stronger than the random variation in initial conditions. However, the only way to assess this risk is by checking the final coherence of the results and the similarity between replicates. In the ENEX experiment, which dealt with a P-limited planktonic community, we expected an increase in primary productivity across the P-enrichment gradient and no significant productivity changes with N imbalance. Protist community composition changes were more unpredictable, but we expected similar responses between treatment replicates. Indeed, there was a coherent general response across the P- and N-addition gradients and similarity in the replicates (Giménez-Grau *et al*., 2020).

**Fig. 2:**
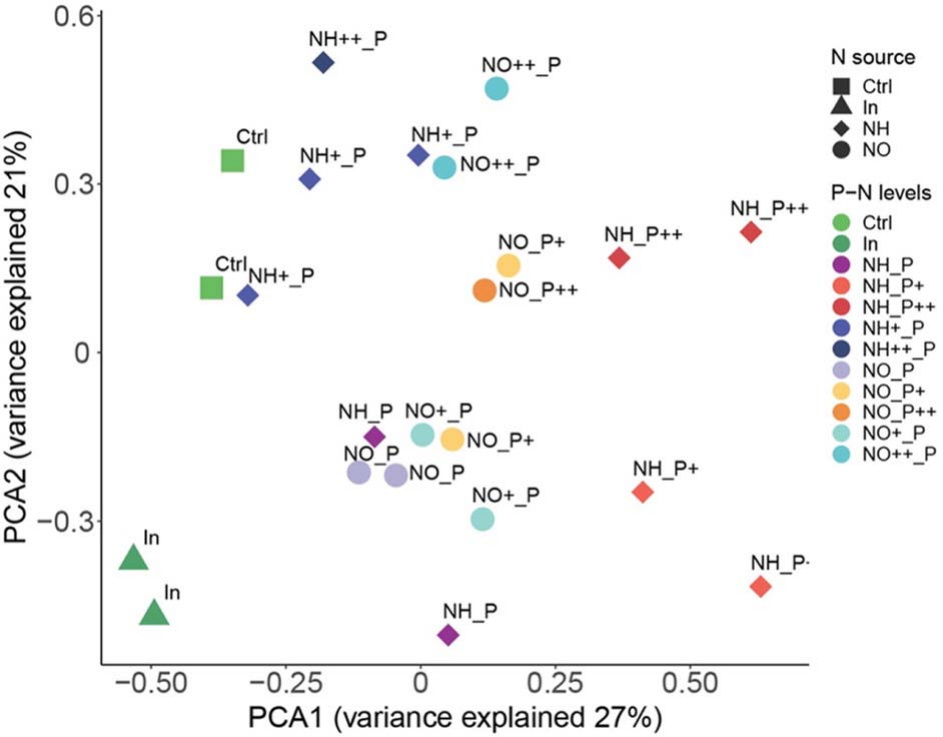
Ordination of the protist species composition in the treatment enclosures of the ENEX experiment using a principal component analysis with the Hellinger distance (Legendre and Gallagher, 2001). Treatment codes and characteristics are indicated in Table II. The first axis mostly discriminates between phosphorus treatment levels, and the second axis nitrogen additions. See Zufiaurre *et al*. (2021) for details on species composition. Note the proximity between replicas.

It may be argued that these general patterns may have been obtained with other methods, for instance, incubation flasks. Although these may be true, the enclosure system provides a more robust result because it minimizes the potential cell damage during manipulation than field or lab flask assays. Despite being an artificial system, it is still much closer to the real world than the lab’s small flask assays. In flask experiments, water must be obtained either from single depths or integrated across the water column and mixed. In the first case, many samples are required to view the photic zone comprehensively; in the second, plankton populations are subjected to substantial stress. In the enclosure system, the haphazard initial differences during filling could be expected to have more influence on the species composition than on the bulk phytoplankton (Chl) growth. However, it was not the case in the ENEX experiment, and the changes in composition were also coherent between replicates (Zufiaurre *et al*., 2021). This coherence is shown by the close position of the replicate pairs in an ordination of the protists’ composition in that experiment (Fig. 2).

**Table II:** Nutrient initial treatment values that defined gradients of P-enrichment and N-imbalance in the ENEX experiment

Gradient	Treatment	TDP	NO_3_^−^	NH_4_^+^	DIN	DIN:TDP
		μmol L^−1^	μmol L^−1^	μmol L^−1^	μmol L^−1^	(a/a)
P-enrichment	NO_P++	1.9	16.8	0.2	17	9
P-enrichment	NH_P++	1.9	4.2	12.8	17	9
P-enrichment	NO_P+	0.21	16.8	0.2	17	81
P-enrichment	NH_P+	0.21	4.2	12.8	17	81
P-enrich/N-imbal	NO_P	0.06	16.8	0.2	17	283
P-enrich/N-imbal	NH_P	0.06	4.2	12.8	17	283
N-imbalance	NO+_P	0.06	34.8	0.2	35	583
N-imbalance	NH+_P	0.06	4.2	30.8	35	583
N-imbalance	NO++_P	0.06	72.8	0.2	73	1217
N-imbalance	NH++_P	0.06	4.2	68.8	73	1217
Control/Initial	NA	0.022	4.2	0.2	4.4	200

The two gradients combined resulted in two similar N:P stoichiometric gradients, differing in the N primary source (Giménez-Grau *et al*., 2020). The control values indicate the average characteristics of the initial filling water

### The added value of the sediment trap

The enclosures are intended for before/after experiments. We may wonder if what is found at the end corresponds to the primary response to the treatment or, perhaps, the experiment has been too long, and we are sampling a decaying phase of the community response. The comparison between the material accumulated in the sediment traps and the material remaining in the water column is highly informative. In the ENEX experiment, the amount of particulate organic matter in the water column of the enclosures was two orders of magnitude higher than in the sediment traps, thus indicating that most of the initial response had not been sedimented.

**Fig. 3:**
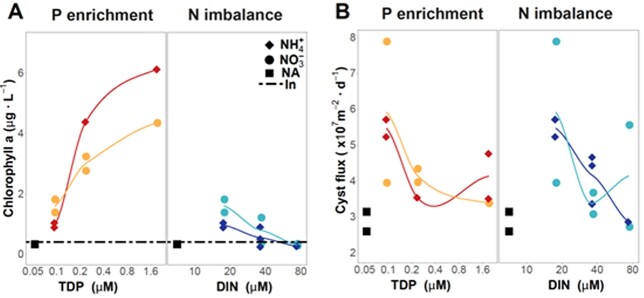
Effects of P enrichment and N imbalance on chlorophyll-a levels (**A**) and chrysophyte cyst fluxes (**B**) during the ENEX experiment. The sediment trap provides a complementary view to the bag. Details of the treatments are indicated in Table II. Replicates are plotted individually except in the case of the initial conditions (In), for which the average value is shown as a linear reference. Note that controls (NA) and the samples for the lowest P and N additions are the same in the P enrichment and N imbalance gradients. Red and dark blue refer to treatments with ammonium as the nitrogen source, and yellow and light blue to treatments with nitrate.

Chrysophytes are the dominant group in the phytoplankton of Lake Redon (Felip *et al*., 1999). These organisms produce resistant silica cysts for which specific catalogs have been developed (Pla, 2001) to study their sediment records and ecological indicator value (Pla-Rabes and Catalan, 2011). The sediment traps at the end of the enclosures allow evaluation of the response to the nutrient enrichments in terms of the cyst fluxes and composition. The chrysophytes’ cyst fluxes in all the additions were higher or similar to the control enclosures (Fig. 3). However, the response patterns along the gradients differed from those shown by Chla. The most significant fluxes were found in the low-addition, implying that some other phytoplankton groups were responsible for the Chla increase observed. In fact, there was an increase in cryptophytes, diatoms, and chlorophytes at higher nutrient levels (Zufiaurre *et al*., 2021).

**Fig. 4:**
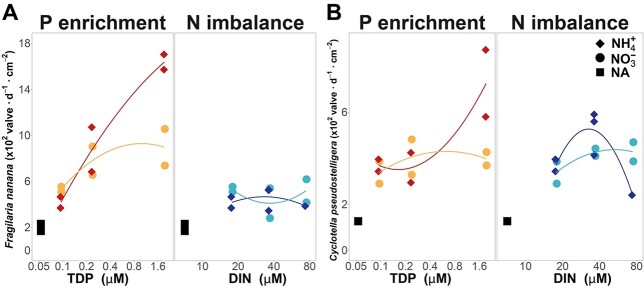
Sedimentation fluxes of the two main diatom species growing during the ENEX experiment evaluated with the sediment traps at the enclosure’s bottom. (**A**) *Fragilaria nanana*, (**B**) *Cyclotella pseudostelligera*). The sediment trap helps to assess the response of rapidly sinking organisms.

Although the enclosure characteristics are not appropriate for maintaining diatoms in suspension, there was a significant growth of *Fragilaria nanana*, a small and thin life form, in higher P treatments of the ENEX experiment. In the initial community, another planktonic diatom, *Cyclotella pseudostilligera*, was relatively abundant; however, this species apparently did not proliferate within the enclosures in any treatment according to the specimens accounted for in the water column of the enclosures. However, we found that the patterns of the two species in the sediment traps were quite similar (Fig. 4). Therefore, *Cyclotella* was actually responding to the treatments but sank much faster than *Fragilaria,* leaving an apparent negative growth in the bags. The ratio between the two species in the sediment traps indicated that *F. nanana* grew relatively better in P-enriched treatments than *Cyclotella* and, on the contrary, showed more inhibition by N excess. Previous field observations had shown that these two species grew during the period of the thermocline deepening in Lake Redon, when mixing starts to contact the first fine sediment layers, increasing nutrients in the water column: *Fragilaria* peaked in September and was followed by *Cyclotella* in October (Catalan *et al*., 2002). The enclosure experiment confirmed a quick positive response of the two species to P increase in oligotrophic waters. Without the sediment trap, we would conclude opposed responses for the two species.

### The enclosure intrinsic effects

The enclosure tubes have a large surface-to-volume ratio that markedly modifies the hydrodynamic conditions, and the plastic bags affect light conditions. Hydrodynamic conditions are always far from natural conditions in enclosures and mesocosms. Artificial mixing in enclosures usually results in hydrodynamic conditions that may be as unrealistic as no mixing (Riebesell *et al*., 2010). In fact, there is an inherent problem in generating turbulent spectra comparable to those occurring in lakes and oceans in any enclosure or laboratory experiments. The characteristic length within which energy dissipation is averaged is much shorter in the enclosures.

Consequently, either the rate of energy dissipation is too high or, if realistic, the Reynolds number (inertial forcing against viscosity) is too low (Osborn, 1996; Catalan, 1999). In the enclosures described, if the wind induces some lake currents, the flexible walls and the long and thin shape of the enclosures can gently stir the inner water content (Gust, 1977). However, it will remain under-mixed relative to the external water or, at least, differently mixed. If the community (e.g. diatom dominance) or the topic to be studied [e.g. grazing rates (Marrase *et al*., 1992)] are highly dependent on mixing conditions, the enclosure described does not appear as a good option.

The light environment is also greatly modified by the enclosure walls. The relative irradiance gradient across the photic zone may be maintained, but the light quality is markedly modified because of the absorption properties of the bag material. In the ENEX experiment, the polythene walls drastically reduced the high UV radiation levels existing in high mountain lakes (Sommaruga, 2001). Indeed, preserving UV radiation in field experiments is not easy and requires low UV-absorbing materials (Halac *et al*., 1997). Furthermore, polythene reduces photosynthetic active radiation (PAR) between 20% and 30% depending on wavelength (Cutroneo and Torrisi, 2014). Therefore, light conditions are drastically modified. One positive aspect is that the large surface-to-volume ratio provides homogenous light conditions at each depth within the enclosure (Berg *et al*., 1999).

The controls, enclosures without additions, account for the enclosure effect. In the ENEX experiment, the first axis of variation shows that control enclosures were the most similar to the initial communities at the end of the experiment (Fig. 2), indicating that enclosure effects were not playing a determinant conditioning effect upon the primary response. However, initial and control samples differed markedly concerning the second axis, probably summarizing the significant environmental change within the enclosure. Only a few species were strongly negatively affected in all treatments (<5%). They included species with contrasting life forms, such as the chrysophycean, *Pseudokephyrion inflatum*, and the ciliate, *Askenasia acrostomia*. Most species grew in many treatments (Zufiaurre *et al*., 2021). In any case, the enclosure effect can be accurately evaluated because of the possibilities of high replication.

### Large zooplankton

As mentioned, the volume and duration of the experiments with these enclosures are appropriate for protist community dynamics (i.e. phytoplankton and small zooplankton) (Parsons, 1982). Large zooplankton could be a source of undesired variation between experimental treatments. The small diameter of the enclosures and the frontal turbulence generated during the uprising filling should partially exclude large zooplankton that shows avoidance reaction to turbulence (Franks, 2001). The degree of avoidance may be different for each species. In the ENEX experiment, three crustacean species were present in the lake plankton community: *Cyclops abyssorum, Diaptomus cyaneus* and *Daphnia pulicaria*. Comparing the outside concentration with the average inside the enclosures indicated a marked avoidance difference between the three species. On average, the inside *Diaptomus* density was <10% than outside, *Daphnia* about 60%, and *Cyclops* showed no significant decrease. In fact, because of the low numbers, the variation between enclosures was high. In four enclosures, we did not find any adult crustaceans (~20%), <50 individuals in 62% of the enclosures, <100 in 90% and <300 individuals in all of them. The differences were mainly due to *Cyclops*, in which juvenile and adults are mainly predators of *Daphnia* in this lake (Ventura and Catalan, 2008). In addition to different avoidance reactions, the patchy nature of the populations in the water column may add some noise. Despite the variation, no significant influence of large zooplankton composition on the results obtained was found.

The zooplankton exclusion can be enhanced by inserting a mesh in the frontal opening of the enclosure. However, the finer the mesh, the larger the risk of excluding other organisms. This is an aspect that any experimental method has to face and handle accordingly with the particular characteristics of the community studied and the experiment goals. Large zooplankton has to be considered a random factor in the enclosures described. Studying multitrophic interactions, including tertiary trophic levels, ideally requires large volumes (>100 m^3^) and probably longer experiments (Riebesell *et al*., 2010).

### Fouling and elemental mass balance

One of the particular features of the enclosures described is the high aspect ratio (wall area vs. water volume). This feature introduces the risk of becoming an experiment on benthic colonization if the duration is too long or some treatments stimulate rapid wall colonization. Adjusting the experiment to short periods minimizes the undesired effects of periphyton growth on the enclosure surfaces (Chen *et al.*, 1997). Wall growth depends not only on the enclosure design and the duration of the experiment but also on the trophic state and size of the water body. Open waters hold fewer benthic organisms as potential inoculum for the enclosure walls than waters in the littoral zone. In the ENEX experiment, we did not observe any significant development of microeukaryotic biofilms on the surface of the bags after a microscope inspection at the end of the experiment, except for spots of zygnematophyceae filaments (i.e. *Spirogyra*) in the inner part of the treatments with the highest P-addition. These algae are common in the littoral epilithon of the lake. The growth and influence of bacterial biofilms are more difficult to be evaluated by direct inspection unless they are huge.

**Fig. 5:**
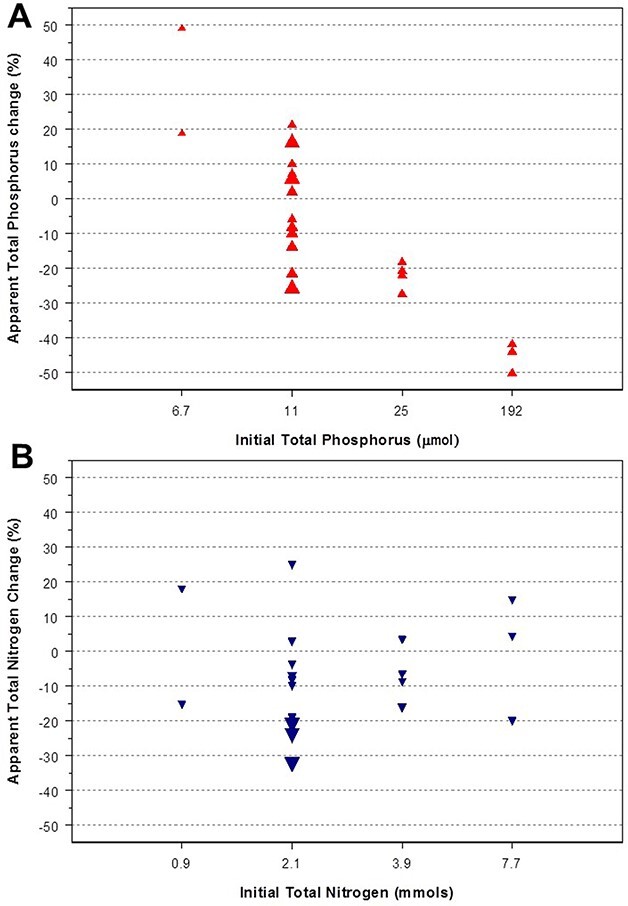
Balance between the initial and final total phosphorus (**A**) and total nitrogen (**B**) in the enclosures. Nitrogen shows a random variation, while the phosphorus pattern indicates some bias at high P additions, probably related to some adsorption to the plastic walls. Initial values were estimated based on two 20-m integrated samples in the lake water column at the beginning of the period. Therefore, the exact initial values in each enclosure could differ. Negative values indicate lower amounts at the end of the period. The size of the symbols shows the initial level of the complementary element, TN in A and TP in B.

Elemental mass budgets are a way to evaluate some biases deriving from the material characteristics and experimental method. In the ENEX experiment, we compared the total initial and final elemental amounts of the nutrients manipulated, P and N (Fig. 5). Although the average difference between initial and final elemental inventories was low, the variation among replicates was relatively large (mean ± standard deviation; P, 8% ± 24; N 7% ± 14). A significant bias in one of the controls (Fig. 5A) was in the positive direction; more P was estimated at the end than at the beginning, which is not possible. This paradox was due to the assumption that the initial TP in the bags was identical to the integrated measure in the lake water column at the filling time, which could be correct on average, but deviations in each enclosure existed, being extreme in the specific case of that control. Because of the added P in other treatments, the deviations from the assumption were only noticeable in the controls.

In the P case, the budget deviations indicated a systematic bias at high initial TP (Fig. 5A), whereas N deviations were random (Fig. 5B). The random error in these budgets was related to the assumption of a common initial composition, which is not exact, and the many fractions analyzed, each with their analytical error. The N case indicates that deviations lower than ±20–30% could be considered potentially random, which was also the case for the low P additions. The high negative P budgets at elevated P additions (Fig. 5B) could be due to the P adsorption to the polythene walls (Hassenteufel *et al*., 1963) and the previously mentioned fouling growth. Whatever the reason, the experiment would have to be shorter for the high P-addition treatments. However, despite the anomalous P budget in the highest P additions, no benthic organisms were found in the microscopy counting of the plankton samples (Zufiaurre *et al*., 2021). The concentrations of TDP at the end of the experiment were still high (~390 nM) in those high P addition enclosures, not limiting the growth. Therefore, the results were reliable. Any nutrient addition experiment should compare total nutrient budgets between initial and final conditions.

### Alternative designs

The enclosure design described allows modifications according to the system to be studied and the purpose of the experiment. For instance, the length of the bags can be shortened for experiments in shallow lakes or thinner photic layers. In these cases, transparent material (e.g. methacrylate) can be used for the upper tube to minimize light reduction. The diameter can also be wider: 20–30 cm appears manageable for transport and deployment from small boats. Polythene bag rolls are still commercially available with these diameters. The upper tube should accommodate the new dimensions, but the sediment trap can be kept as currently designed, just placing an appropriate funnel connecting the bag and trap. Changing the bag material can also be helpful for experiments on light/dark expositions or investigating irradiance effects. It can also be considered to sample the internal gradient that develops within the long bags rather than integrate it. Another option could be modifying the bottom tub for benthic-pelagic coupling experiments (Dimitriou *et al*., 2017). Application of the system in marine conditions is also feasible, probably, enhancing the robustness of the construction and planning for short-duration deployments during forecasted good weather.

## CONCLUSIONS

The enclosure type described is an addition to the experimental toolbox for plankton investigation. The enclosures are particularly suitable for studying the immediate response of the planktonic community to pulse perturbations of nutrients, or other dissolved substances, using before and after comparisons. The design offers the possibility of obtaining an integrated assessment across the entire or most of the photic zone, facilitating multiple replicates and treatments. Although the enclosures were initially thought for experiments in remote oligotrophic systems, the design can be easily tailored and expanded for applications to a large variety of water masses, maintaining its essential differential features: low perturbation of the planktonic organisms during filling and possibilities for high replication.

## Supplementary Material

enclosures_fbac074

## Data Availability

The ENEX experiment data is available in the Dryad Digital Repository, doi: 10.5061/dryad.tx95x69vh (Catalan et al., 2020).
